# Editorial: Artificial intelligence education & governance -human enhancive, culturally sensitive and personally adaptive HAI

**DOI:** 10.3389/frai.2024.1443386

**Published:** 2024-07-01

**Authors:** Rajiv Kashyap, Yana Samuel, Linda Weiser Friedman, Jim Samuel

**Affiliations:** ^1^William Paterson University, Wayne, NJ, United States; ^2^Middlesex County College, Edison, NJ, United States; ^3^Baruch College (CUNY), New York, NY, United States; ^4^Rutgers, The State University of New Jersey, New Brunswick, NJ, United States

**Keywords:** artificial intelligence, education, governance, personally adaptive, culturally sensitive, human enhancive, human centered, AI ethics

“*AI can advance in ways that support, rather than erode, fundamental human aspirations”*

Shneiderman (2022)

A new era of artificial intelligence has begun, wherein artificial intelligence (AI) has emerged as a dominant societal paradigm that increasingly influences nearly every sphere of human life (Samuel et al., 2024a). While AI holds great promise, it also gives rise to hitherto unidentified problems and uncertainties - the emerging complexities of socio-technical challenges associated with human-like AI are increasing and are not expected to be resolved in the foreseeable future (Brynjolfsson, 2022). Extant research posits that the broad and explosive development of AI technologies, while advantageous, is also fraught with risks and the emergence of sophisticated new threats across domains such as medicine, education, law and governance, and military, among others (Hashimoto et al., 2018; Jensen et al., 2020; Köbis et al., 2022; Hendrycks et al., 2023; Park et al., 2023). While technological revolutions are often marked by chaos, confusion, and fear, these challenges have been amplified by a combination of the unprecedented potential for rapid transformation of human society and the fragmented, and often AI-phobic public information about AI (Samuel et al., 2024b). To counter the potentially destabilizing effects of AI on society, it is necessary to establish research, policy, education, and practice initiatives that avoid harms and minimize the risks associated with the deployment of AI technologies. Additionally, we must ensure that we preserve the core values that guide individuals, cultures, and societies while supporting rapid advancements in AI. As the famous philosopher Alfred N. Whitehead eloquently stated, “The art of progress is to preserve order amid change and to preserve change amid order,” reminding us of the delicate balance needed during times of rapid innovation.

Fortunately, along with the remarkable progress in the development of AI applications, we are witnessing the emergence of an essential and complementary paradigm: Human-centered AI (HAI). HAI, also known as HCAI, has been gaining attention as we grapple with the uncertainties and complexities of human agency in an AI driven future (Shneiderman, 2022). Although previously conceptualized in a variety of ways, HAI appears to be converging toward frameworks that posit AI augmented human performance and AI supported human behavior (Pelaez et al.; Samuel et al., 2022; Samuel et al.). We believe that HAI is a critical paradigm in the evolution of AI that must be aligned with human values and societal goals to be beneficial and sustainable. In our view, HAI embodies three key dimensions: *human enhancive, culturally sensitive and personally adaptive*.

*Human enhancive* is a human-first ideology that underpins the development of Human-Centered AI (HAI) applications. It ensures that all human-AI interaction designs prioritize human wellbeing. *Culturally sensitive* refers to the need to ensure that AI, and generative AI in particular, possesses fine-tuned or customizable capabilities that can shape the experiences of users and user-groups with specific sociocultural needs (Samuel et al.). *Personally adaptive* refers to the quantifiable improvements in AI-driven performance that emanate from AI driven adaptations for individualized or group support and extend to broader applications. Initially focusing on enhancing individual performance, personally adaptive AI technologies can be scaled to benefit groups, communities, and organizations, thereby maximizing their overall effectiveness and efficiency (Samuel et al., 2022). Our paper Samuel et al. titled “*Cultivation of human-centered artificial intelligence: culturally adaptive thinking in education for AI*” embodies the principles of HAI. Our CATE-AI framework draws attention to designing AI education to enhance human capabilities and augment human performance through a culturally sensitive AI education framework. We emphasize the need for culturally responsive teaching and cultural intelligence to ensure that AI education is relevant and effective across diverse cultural contexts. CATE-AI is personally adaptive to individual sociocultural needs and offers ways of understanding how AI learning experiences can be tailored to increase understanding and engagement. HAI is the theme that undergirds the papers in this Research Topic as we discuss below.

## Human enhancive

In their article titled “*Human-centricity in AI governance: A systemic approach*” Sigfrids et al. highlight the human-enhancive potential of AI by focusing on inclusive and comprehensive governance frameworks that prioritize human values and societal wellbeing. They underscore the importance of mutual trust, transparency, and communication as foundational elements for socially sustainable AI deployment. Shifting focus to educational applications, Ognibene et al. discuss the creation of a social media virtual companion aimed at educating and supporting teenage students navigating social media. In their article titled, “*Challenging social media threats using collective well-being-aware recommendation algorithms and an educational virtual companion*,” they suggest that humans can enhance collective wellbeing by co-opting experts and educators in interactions to benefit the community. Expanding on such educational advancements, van Leeuwen et al. emphasize a human-enhancive design philosophy by involving teachers as the primary stakeholders in the design of AI systems in education. Their article titled, “*Participatory design of teacher dashboards: navigating the tension between teacher input and theories on teacher professional vision*” seeks to ensure that AI tools, such as teacher dashboards, are tailored to the specific needs and practices of educators. Leveraging the concept of the well-known “Turing test,” Pelaez et al. in their article titled “*The Turing teacher: identifying core attributes for AI learning in K-12*,” discuss the potential for humans to enhance the use of AI in K-12 settings through the concept of a “Turing Teacher” that can facilitate learning and address the diverse needs of students. Moving toward personalized learning experiences, Sumi and Sato's article titled “*Experiences of game-based learning and reviewing history of the experience using player's emotions*” exemplifies HAI by discussing the potential to enhance learning through personalized and emotionally engaging experiences. In a similar vein, Schmitz Hubsch et al. in their article titled “*Affective response categories—toward personalized reactions in affect-adaptive tutoring system*s” focus on tailoring educational experiences based on individual emotional states. They highlight the potential to enhance the learning process by recognizing and responding to the unique emotional and cognitive needs of each learner. To further augment learner engagement and motivation, Dermeval et al. introduce the Gamification Tutoring Ontology (GaTO) in their article titled “*GaTO: An ontological model to apply gamification in intelligent tutoring systems*.” This ontological model integrates gamification into Intelligent Tutoring Systems (ITS), thereby enriching human learning experiences.

## Culturally sensitive

Sigfrid et al.'s call for integrating community and society-centered perspectives into AI governance underscores the culturally sensitive dimension of HAI. They emphasize the importance of including diverse societal perspectives in AI governance frameworks to ensure culturally sensitive deployment of AI technologies. Transitioning to educational contexts, van Leeuwen et al. paper reflects the culturally sensitive aspect of HAI by recognizing the tension between stakeholder input and educational theory. They advocate for a balanced integration to achieve effective and pedagogically sound AI solutions, promoting the personally adaptive nature of HAI by focusing on the development and use of diagnostic cues and diagnostic tools. Ognibene et al. framework is mindful of cultural diversity, emphasizing the importance of community involvement in setting desirable conditions and crafting educational content that respects and mirrors a range of cultural viewpoints. Similarly, Pelaez et al. emphasize the need to be culturally sensitive to the challenges of using AI to close the digital divide in traditionally disadvantaged communities. Sumi and Sato further stress the need for cultural sensitivity by adapting learning scenarios to be relevant and inclusive across diverse cultural contexts. Their work highlights the necessity of creating learning environments that accommodate and respect cultural differences, ensuring inclusivity in educational experiences. Real-time recognition and adaptation to user emotions and actions allow for a highly personalized educational experience tailored to individual learning styles (Dermeval et al.). The GaTO model incorporates motivational needs and learning preferences across various cultural contexts to ensure that ITS designs are culturally sensitive.

## Personally adaptive

Sigfrid et al. emphasize the importance of adaptive governance models that respond to the evolving needs and values of different stakeholders, describing how AI can be tailored to enhance individual and collective human capabilities. Building on the theme of personalized education, Ognibene et al. propose that an AI-powered recommendation system can be scaled to enhance collective wellbeing through personalized educational experiences adapted to individual requirements. This notion is further supported by Pelaez et al., who highlight the significance of collaboration between AI technologies and real teachers, emphasizing the need to coordinate and adapt AI tools to meet the specific needs of students. This approach positions AI not merely as a tool but as a partner in the educational process. Sumi and Sato emphasize the potential for dynamic adaptation by advocating the use of AI tools that can recognize and respond to user emotions and actions in real-time. This capability allows for a highly personalized educational experience tailored to individual learning styles, thereby making learning more inclusive and effective. Extending this concept, Schmitz Hubsch et al. demonstrate a commitment to culturally sensitive AI design, incorporating diverse emotional expressions and reactions that may vary across cultural contexts. Ensuring that AI evolves with the user's changing emotional and cognitive states highlights its alignment with the personally adaptive dimension of HAI. Continuing with the focus on personalized learning, Dermeval et al. introduce the GaTO model, which underscores the importance of personalizing tutoring strategies based on individual learner behaviors and interactions. This ensures that educational content and methods evolve in response to the unique needs of each student, thereby enriching human learning experiences.

These papers also highlight the need for multifaceted and multidisciplinary approaches to prepare human intelligence for AI-driven performance enhancement. In tandem with developing human-interactive AI applications such as translation and handwriting (Jain et al., 2023; Anderson et al., 2024), it is crucial to address issues like bias in AI and the AI discipline. In addition, other challenges related to human-oriented AI must be explored in future research (Silberg and Manyika, 2019; Samuel et al., 2020; Borenstein and Howard, 2021). By embracing Human-Centered AI, we can ensure that AI technologies advance in ways that support and enhance fundamental human aspirations, aligning technological progress with societal values and goals.

Collectively, the papers in this Research Topic provide a critical foundation to transition research and transform society into the next phase of evolving human-supportive AI: the agentic AI paradigm. By integrating human-centered, culturally sensitive, and personally adaptive approaches, the authors in this Research Topic have laid the groundwork for research on advanced AIs and autonomous AI agents that can operate independently while prioritizing human values and societal goals. HAI driven transformation will enhance our ability to interact with and adapt to complex AI environments, while advancing the capabilities of AI technologies effectively, responsibly and ethically.

## Author contributions

RK: Writing – original draft, Writing – review & editing. YS: Writing – original draft, Writing – review & editing. LF: Writing – original draft, Writing – review & editing. JS: Writing – original draft, Writing – review & editing.
